# Supplementary material for: Content-rich biological network constructed by mining PubMed abstracts
Source: BMC Bioinformatics. 2004 Oct 8;5:147. doi: 10.1186/1471-2105-5-147 (PMC528731; doi:10.1186/1471-2105-5-147)
Supplement: Additional File 2 — The original results of the above study (non-essential files are deleted to keep the file size under the limit set by BMC bioinformatics). [file 1471-2105-5-147-S2.bz2 › chilibotAdditionalFile2/dip05/436ID7545673E10381/html/CCR1_CCL7.html]

 


 **CCR1** and **CCL7** 
  
Found 54 abstracts in PubMed, retrieved 05.  
 

 What does Google say? 
 PDF only 
| .edu only 

---

**Interactive relationship** (e.g. stimulation, inhibition, etc)

**Neutral relationship**- Expression of L CCR  [ **CCR1** ]  in HEK 293 cells reveals functional responses to CCL2, CCL5,  **CCL7** , and CCL8.  Ref: 12885941 J Leukoc Biol, 2003

**Non-interactive relationship** (e.g. studied together, co-existance, homology, etc.)

- We used radioligand binding and 35S GTPgammaS exchange assays in membranes from a cell line transfected to express  **CCR1**  Ba F3 hCCR1 to characterize a panel of chemokines HCC 1, MIP 1alpha, MIP 1beta, MIP 1delta, MPIF 1, MCP 2, MCP 3  [ **CCL7** ] , and RANTES as  **CCR1**  ligands.  Ref: 12381680 Br J Pharmacol, 2002
